# Aspiration-Related Acute Respiratory Distress Syndrome in Acute Stroke Patient

**DOI:** 10.1371/journal.pone.0118682

**Published:** 2015-03-19

**Authors:** Jiang-nan Zhao, Yao Liu, Huai-chen Li

**Affiliations:** Department of Respiratory Medicine, Provincial Hospital Affiliated to Shandong University, Jinan, Shandong, 250021, China; University of Bari, ITALY

## Abstract

**Background:**

Aspiration of oral or gastric contents into the larynx and lower respiratory tract is a common problem in acute stroke patients, which significantly increases the incidence of acute respiratory distress syndrome (ARDS). However, little is known about the clinical characteristics of aspiration-related ARDS in acute stroke patients.

**Methods:**

Over 17-month period a retrospective cohort study was done on 1495 consecutive patients with acute stroke. The data including demographic characteristics, clinical manifestations, laboratory examinations, chest imaging, and hospital discharge status were collected to analysis.

**Results:**

Aspiration-related ARDS was diagnosed in 54 patients (3.6%). The most common presenting symptom was tachypnea (respiratory rate ≥25 breaths/min) in 50 cases. Computed tomography (CT) images usually demonstrated diffuse ground-glass opacities (GGOs) and inhomogeneous patchy consolidations involving the low lobes. Age, NIHSS score, GCS score, dysphagia, dysarthria, hemoglobin concentration, serum aspertate aminotransferase (AST), serum albumin, serum sodium, and admission glucose level were independently associated with aspiration-related ARDS (odds ratio (OR) 1.05, 95% confidence interval (CI) (1.04–1.07); OR 2.87, (2.68–3.63); OR 4.21, (3.57–5.09); OR 2.18, (1.23–3.86); OR 1.67, (1.31–2.14); OR 2.31, (1.11–4.84); OR 1.68, (1.01–2.80); OR 2.15, (1.19–3.90); OR 1.92, (1.10–3.36) and OR 1.14, (1.06–1.21) respectively).

**Conclusions:**

Aspiration-related ARDS frequently occurs in acute stroke patient with impairment consciousness. It is advisable that performing chest CT timely may identify disease early and prompt treatment to rescue patients.

## Introduction

Every year in the worldwide, approximately fifteen million people experience a new or recurrent stroke and up to two-thirds of victims suffer permanent disability or death [1,2]. Evidence have shown that medical complications are the major causes of morbidity and mortality after acute stroke [2,3]. Aspiration of oropharyngeal or gastric contents into the larynx and lower respiratory tract is a common problem in acute stroke patients due to swallowing difficulty, neurologic dysphagia and gastrointestinal dysfunction [4,5]. Aspiration induced lung injury accounts for a significant proportion of mortality in acute stroke patients [2,3,6].

In daily clinical practice, the major pulmonary syndromes caused by aspiration in acute stroke patients are frequently considered to be bacterial infection. Despite of taking reasonable and effective antibiotic therapy and high-flow oxygen therapy, there is still a high occurrence of unsolved hypoxia and mortality. In fact, aspiration after acute stroke can cause several dreaded medical complications, such as aspiration pneumonitis, airway obstruction, exogenous lipoid pneumonia, diffuse aspiration bronchiolitis, and aspiration pneumonia with or without lung abscess [6–8]. The most severe complication is ARDS, which is characterized by acute respiratory failure with severe hypoxia and diffuse pulmonary infiltrates leading to longer hospitalization, poorer quality of life and higher mortality [9,10].

Previous studies have identified aspiration is a major direct cause of ARDS [9–11], but little is known about the clinical and imaging characteristics of the aspiration-related ARDS. Ascribing ARDS to aspiration may be challenging. The early diagnosis of aspiration pneumonia is crucial. The present investigation was conducted with the following aims: first, to elucidate the clinical and radiological features of aspiration-related ARDS in acute stroke patients; second, to determine risk factors for the disease to early prevention; and third, to aid physicians in the early diagnosis and treatment to improve the outcomes.

## Methods

### Ethical Approval

The study was approved by the Ethic Committee of Shandong Provincial Hospital, China. Written informed consent was obtained from all participants or their surrogates.

### Data collection and definition

The study was based on a stroke registry in China, which is a prospective registry of consecutive patients with acute stroke. Our study is a retrospective study about acute stroke patients in the registry. The patients were selected from Shandong Provincial Hospital, a tertiary-care, university-affiliated hospital. All data were extracted from the electronic medical record (EMR) system by trained research coordinators.

To be eligible for this study, all acute stroke patients had to meet the following criteria [1–4]: (1) aged 18 years or older; (2) diagnosed according to the World Health Organization definition as rapidly developed clinical signs of cerebral function disturbance, of a vascular origin, and classified based on results from first brain scan into cerebral infarct, intracerebral hemorrhage, and subarachnoid hemorrhage; (3) presented within 24 hours of the onset of acute stroke; (4) confirmed by head computerized tomography (CT) or brain magnetic resonance imaging (MRI).

**Data collection.** For the present study, the followings were analyzed: (1) demographics (age, gender, et al), (2) clinical symptoms on admission (dysphagia (documented by a standardized dysphagia screening test), dysarthria, et al) and new symptoms and signs after admission (fever, dyspnea, tachypnea, et al); (3) chest radiological findings; (4) stroke risk factors: hypertension (any treatment and/or patient’s self-report), diabetes mellitus (any treatment and/or patient’s self-report), atrial fibrillation (patient’s self-report and/or documented by standard electrocardiogram), coronary heart disease, smoking history, and excess alcohol consumption (≥2 standard alcohol beverages per day); (5) preexisting comorbidities: congestive heart failure, valvular heart disease, chronic obstructive pulmonary disease (COPD), peptic ulcer, previous gastrointestinal bleeding (GIB), renal failure, cancer, et al; (6) National Institutes of Health Stroke Scale (NIHSS) score on admission, Glasgow Coma Scale (GCS) score on admission, and Acute Physiology and Chronic Health Evaluation II (APACHE II) score for patients with ARDS; (7) mechanical devices (nasogastric tube, invasive/non-invasive mechanical ventilation used after admission for unsolved hypoxia despite of high-flow oxygen); (8) laboratory indices on admission; (9) hospital length-of-stay (LOS) (days); (10) hospital discharge status (survive or decease).

**Definition of aspiration-related ARDS.** Aspiration-related ARDS after acute stroke were diagnosed by treating physicians and prospectively registered by trained reasearch coordinators. Only the disease that developed after hospital admission is registered. Patients had either previously diagnosed aspiration pneumonia according to the following criteria.

Inclusion criteria: (1) Clinical signs and symptoms following an observed episode of vomiting or regurgitation: a sudden worsening of dyspnea within 12h, development of hypoxia with partial pressure of oxygen (PaO_2_) < 90mmHg, abnormal breath sounds, radiographic pulmonary abnormalities (not due to preexisting or other known causes by chest radiography or high-resolution CT), fever or leucocytosis; (2) Either the aspiration had been observed, or gastric contents had been suctioned from the endotracheal tube following intubation; (3) Or requirement for intensive care (defined as the use of mechanical ventilation or vasopressors against shock). However, in many patients the episode of aspiration is not observed, but when the above clinical changes appear suddenly in a patient with previously normal lungs, it is virtually consider that the cause is aspiration.

Exclusion criteria: (1) Nosocomial pneumonia or healthcare-associated pneumonia (HCAP); (2) Severe immunosuppression (HIV, use of immuno-suppressant such as cytotoxic drugs, Cyclosporins, Monoclonal antibodies etc.); (3) A preexisting medical condition with life expectancy less than 3 months (i.e., malignancy).

ARDS is diagnosed by the treating physician based on the Berlin definition [11,13].(Table 1) Aspiration-related ARDS is defined as that developing after aspiration. Other etiologies of ARDS such as sepsis, major trauma, multiple transfusions, pulmonary contusion, and acute pancreatitis were excluded [9,10,12,13]. Pulmonary edema, congestive heart failure, interstitial lung disease, active tuberculosis, radiation pneumonitis, pulmonary infiltration with eosinophilia, widespread infection, pulmonary alveolar hemorrhage alveolar proteinosis, bronchioloalveolar cell carcinoma were also ruled out, in which patients frequently have hypoxia and diffuse pulmonary infiltrates. The chest radiological images were reviewed by two radiologists. After the diagnosis of aspiration pneumonia, all patients were managed by both neurologists and pulmonologists.

**Table 1:** The Berlin definition of acute respiratory distress syndrome.

Timing	Within one week of a known clinical insult or new or worsening respiratory symptoms
Chest imaging^a^	Bilateral opacities—not fully explained by effusions, lobar/lung collapse, or nodules
Origin of edema	Respiratory failure not fully explained by cardiac failure or fluid overload Need objective assessment (eg, echocardiography) to exclude hydrostatic edema if no risk factor present
*Oxygenation* ^*b*^	
Mild	200mmHg<PaO_2_/FiO_2_≤300 mmHg with PEEP or CPAP≥5cm H_2_O^c^
Moderate	100mmHg<PaO_2_/FiO_2_≤200 mmHg with PEEP≥5cm H_2_O
Severe	PaO_2_/FiO_2_≤100 mmHg with PEEP≥5cm H_2_O

Abbreviation: CPAP, continuous positive airway pressure; FiO_2_, fraction of inspired oxygen; PaO_2_, partial pressure of arterial oxygen; PEEP, positive eng-expiratory pressure.

^a^ Chest radiograph or computed tomography scan.

^b^ If altitude is higher than 1000m, the correction factor should be calculated as follows: [PaO_2_/FiO_2_ ×(barometric pressure/760)].

^c^ This may be delivered noninvasively in the mild acute respiratory distress syndrome group.

### Statistical analysis

Categorical variables are summarized as proportions; continuous variables are summarized with mean and standard deviation (SD). In univariate analysis, *X* ^*2*^ test is used to compare categorical variables, and one-way ANOVA is used to compare continuous variables. *X* ^*2*^ test and one-way ANOVA are used to compare the clinical features and outcomes of the patients with aspiration-related ARDS who survived with those who died. To identify independent risk factors that are associated with aspiration-related ARDS, multivariate logistic regression analysis is used to adjust for confounders. The adjusted odds ratios (OR), the 95% confidence interval (CI) and P value for individual variables are obtained using a logistic regression model, and P < 0.05 is considered to be statistically significant. To assess the discriminatory ability of the model, the *c* statistic is calculated from the logistic regression model predicting aspiration-related ARDS. The *c* statistic, which represents the area under the receiver operating characteristic (ROC) curve, ranges from 0.5 (which indicates no better discrimination than chance) to 1.0 (perfect discrimination). The area under the curve (AUC) and its standard error (SE) are also obtained. All analyses are performed with SPSS version 17.0 for Window.

## Results

### Demographic characteristics

Patients’ characteristics are shown in Table 2. From January 2012 to May 2013, a total of 1495 patients with acute stroke were admitted to the study. Their mean age was 62 years (mean±SD, 61.7±13.5 years) and 69.6% were male, and 109 patients died with a mortality rate of 7.3%. Among the 1495 patients with acute stroke, mechanical ventilation (MV) was required in 64 (4.3%) patients, including invasive (n = 40, 62.5%) and non-invasive (n = 24, 37.5%).

**Table 2:** Characteristics in the groups with aspiration-related ARDS and no pulmonary complication: results of univariate analysis.

	Total sample N(%)	Aspiration-related ARDS N(%)	Aspiration pneumonia without ARDS N(%)	No pulmonary complication N(%)	P value
Number of patients	1495	54	359	1082	
Age (year) (mean±SD)	61.7±13.5	66.0±13.2	64.7±14.0	60.2±13.2	<0.001
Male gender	1040(69.6)	37(68.5)	239(66.6)	764(70.6)	0.112
***Acute stroke classification***					
Intracerebral hemorrhage	308(20.6)	14(25.9)	96(26.7)	198(18.3)	0.009
Cerebral infarct	1130(75.6)	38(70.4)	255(71.0)	837(77.4)	0.108
Subarachnoid hemorrhage	55(3.7)	2(3.7)	7(1.9)	46(4.3)	0.054
Smoking	517(34.6)	19(34.8)	124(34.5)	374(34.6)	0.604
Alcohol abuse	208(13.9)	16(29.6)	103(28.7)	361(33.4)	0.062
Admission NIHSS score (mean±SD)	6.4±5.5	9.5±6.2	8.9±5.9	4.5±3.6	<0.001
Admission GCS score (mean±SD)	10.8±4.6	5.6±2.1	6.4±2.9	11.3±3.7	<0.001
Dysphagia	247(16.5)	21(38.9)	137(38.2)	89(8.2)	<0.001
Dysarthria	281(18.8)	15(27.8)	104(29.0)	162(15.0)	<0.001
Choke	272(18.2)	15(27.8)	101(28.1)	156(14.4)	<0.001
***Comorbidities***					
Hypertension	1051(70.3)	39(72.2)	261(72.7)	751(69.4)	0.181
Diabetes	308(20.6)	13(24.1)	85(23.7)	210 (19.4)	0.523
Coronary heart disease	275(18.4)	11(20.4)	72(20.1)	192(17.8)	0.145
Atrial fibrillation	21(1.4)	1(1.9)	8(2.2)	12(1.1)	0.096
Heart failure	32(2.2)	0(0)	11(3.1)	21(1.9)	0.237
COPD	19(1.3)	2(3.7)	10(2.8)	7(0.9)	0.158
***Laboratory values on admission***					
Hemoglobin concentration (g/L) (mean±SD)	127±36	112±39	118±32	137±30	<0.001
Platelet count (×10^9^/L) (mean±SD)	158±68	97±49	101±53	189±82	0.004
WBC count (×10^9^/L) (mean±SD)	7.3±5.0	7.9±5.6	8.5±5.2	7.2±4.7	0.081
Serum AST (U/L) (mean±SD)	43±17	77±28	57±23	21±12	<0.001
Serum albumin (g/L) (mean±SD)	36±13	29±16	33±17	39±11	<0.001
Serum bilirubin (μmol/L) (mean±SD)	18.7±16.2	19.8±14.5	20.1±16.4	16.9±19.5	0.073
Serum creatinine (μmol/L) (mean±SD)	116.2±35.0	137.1±45.4	139.8±43.2	109.3±34.2	0.003
Serum sodium (mmol/L) (mean±SD)	140.5±17.2	135.6±15.2	136.0±16.1	142.7±18.3	<0.001
Admission glucose level (mmol/L) (mean±SD)	7.0±2.8	8.2±3.6	8.8±3.7	6.5±2.2	<0.001
Admission glucose level >11.0mmol/L	131(8.8)	9(16.7)	58(16.2)	64(5.9)	<0.001
Hospital LOS (days) (mean±SD)	16.9±11.6	20.9±16.2	18.5±11.2	14.7±11.8	<0.001
Mortality	109(7.3)	19(35.2)	41(11.4)	49(4.5)	<0.001

Abbreviation: SD, standard deviation; NIHSS, National Institutes of Health Stroke Scale; GCS, Glasgow Coma Scale; COPD, chronic obstructive pulmonary disease; AST, aspertate aminotransferase; WBC, white blood cell; LOS, length-of-stay.

In total, 54 patients (3.6%) were diagnosed with aspiration-related ARDS, 359 patients were diagnosed with aspiration pneumonia without aspiration-related ARDS, and 1082 patients were no pulmonary complications. The mean days from onset of acute stroke to the diagnosis of aspiration-related ARDS were 9 days (mean±SD, 8.73±3.12 days). The mean age of aspiration-related ARDS was 66 years (mean±SD, 66. ± 13.2 years), and 19 of these patients died with a high hospital mortality rate of 35.2% (n = 19). The mean hospital LOS was 21 days (mean±SD, 20.9±16.2 days) for aspiration-related ARDS.

### Clinical characteristics and radiological findings of aspiration-related ARDS

Table 3 shows the clinical characteristics between aspiration-related ARDS survivors and non-survivors. Patients with aspiration-related ARDS commonly presented with prodromal symptoms, tachypnea (respiratory rate (RR) ≥25 breaths/min) or shortness of breath, followed by rapid progression to respiratory failure with hypoxia (partial pressure of arterial oxygen (PaO_2_)<60mmHg) on room air measured by blood gas analysis. Tachypnea and fever (body temperature (T)≥37.5℃) were recorded in 50 and 28 patients, respectively.

**Table 3:** Clinical characteristics of survivors and non-survivors with aspiration-related ARDS.

Variables	Total N(%)	Survivors N(%)	Non-survivors N(%)	P value
Number of patients	54	35	19	
Age (years) (mean±SD)	66.0±13.2	63.4±13.0	70.2±14.4	0.003
Male gender	37(68.5)	24(68.6)	13(68.4)	0.326
Tachypnea (RR≥25 breaths/min)	50(92.6)	32(91.4)	18(94.7)	0.085
Fever (T≥37.5℃)	28(51.9)	17(48.6)	11(57.8)	0.183
PaO_2_/FiO_2_	220.39±137.61	251.42±105.47	175.92±71.80	0.006
APACHE II score	29.2±19.1	24.4±15.3	36.6±17.5	<0.001
Invasive Ventilator care	38(70.4)	26(74.3)	12(63.2)	0.004
Non-invasive Ventilator care	11(20.4)	7(20.0)	4(21.1)	0.235
*Duration of mechanical ventilation*				
Invasive, (days) (mean±SD)	6.18±5.44	6.78±5.69	5.71±5.02	0.098
Non-invasive, (days) (mean±SD)	7.35 ± 6.47	7.82 ± 6.33	7.24 ± 7.06	0.150
pH value (mean±SD)	7.39± 0.50	7.40± 0.48	7.30± 0.51	<0.001
Acidosis (pH < 7.35)	6(11%)	1(2.9%)	5(26.3%)	<0.001

Abbreviation: SD, standard deviation; RR, respiratory rate; T, temperature; PaO_2_/FiO_2_, partial pressure of arterial oxygen/fraction of inspired oxygen; APACHE II Acute Physiology and Chronic Health Evaluation II.

Of the 54 patients with aspiration-related ARDS, 38 patients were supported by invasive mechanical ventilation, and 11 patients were supported by non-invasive ventilation. Of these 38 patients with IMV care, 26 patients were survived, 12 patients were died. The mean duration of invasive and non-invasive mechanical ventilation was 6 days (mean±SD, 6.18±5.44 days) and 7 days (mean±SD, 7.35±6.47 days), respectively. According to the EMR records, the surrogates of other 5 patients with aspiration-related ARDS refused to use ventilation care and signed the informed consent of refusing treatment.

**Fig 1:**
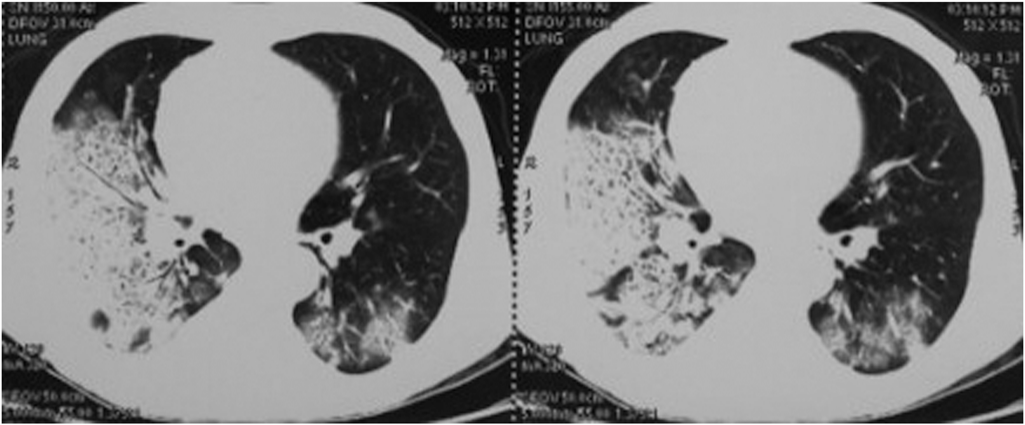
Radiologic finding in a 68-year-old acute stroke man with aspiration-related ARDS.

**Fig 2:**
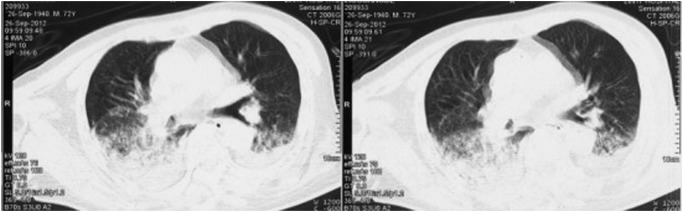
Radiologic finding in a 74-year-old acute stroke man with aspiration-related ARDS.

Chest X-ray usually shows bilateral opacities with infiltration in the lower area. The common features on CT images are widespread GGOs and inhomogeneous consolidations patchy along lung marking distribution in the low lobes. Pleural effusion and air bronchogram also could be observed. Opacities in the upper lung are less common findings. Usually bilateral lungs were involved, and lesions in right lung were severe than the left. The characteristics of CT images were shown in Figs. 1 and 2.

Response to treatment and outcome can be evaluated in patients based on several criteria such as decrease in breathing rate, increase in oxygenation at rest and sleep, and improvement on chest CT images. To identify prognostic factors, the survivors group was defined as those patients who response to active treatment, and non-survivors group was those patients who were died. The age, PaO_2_/FiO_2_, invasive ventilator care, APACHE II score, blood pH value and acidosis were significantly associated with the prognosis (P<0.05). These suggest that early diagnose combined with early supportive treatment is associated with considerable outcome.

### Risk factors

Aspiration-related ARDS were more frequently among patients with older age (P<0.001), intracerebral hemorrhage (P = 0.009), and to have dysphagia (P<0.001), dysarthria (P<0.001), choke (P<0.001). Patients with aspiration-related ARDS also had significantly higher NIHSS scores (P<0.001), serum creatinine levels (P = 0.003), serum aspertate aminotransferase (AST) levels (P<0.001), and blood glucose levels (P<0.001); and lower GCS scores (P<0.001), hemoglobin concentration (P<0.001), platelet counts (P = 0.004), serum albumin levels (P<0.001), serum sodium levels (P<0.001).

On multiple logistic regression analysis, significant risk factors for aspiration-related ARDS are shown in Table 4. The main risk factors (P <0.05) were age (OR 1.05, 95%CI 1.04–1.07), admission NIHSS score (OR 2.87, 95%CI 2.68–3.63), admission GCS score (OR 4.21, 95%CI 3.57–5.09), dysphagia (OR 2.18, 95%CI 1.23–3.86), dysarthria (OR 1.67, 95%CI 1.31–2.14), hemoglobin concentration (OR 2.31, 95%CI 1.11–4.84), serum AST (OR 1.68, 95%CI 1.01–2.80), serum albumin (OR 2.15, 95%CI 1.19–3.90), serum sodium (OR 1.92, 95%CI 1.10–3.36), and admission glucose level (OR 1.14, 95%CI 1.06–1.21).

**Table 4:** Predictors of aspiration-related ARDS—results of multivariate logistic regression analysis.

Variable	0R value	95% CI	P value
Age	1.05	1.04–1.07	<0.001
Admission NIHSS score	2.87	2.68–3.63	<0.001
Admission GCS score	4.21	3.57–5.09	<0.001
Dysphagia	2.18	1.23–3.86	0.008
Dysarthria	1.67	1.31–2.14	<0.001
Hemoglobin concentration	2.31	1.11–4.84	0.026
Serum AST	1.68	1.01–2.80	0.047
Serum albumin	2.15	1.19–3.90	0.011
Serum sodium	1.92	1.10–3.36	0.022
Admission glucose level	1.14	1.06–1.21	<0.001

Abbreviation: NIHSS, National Institutes of Health Stroke Scale; GCS, Glasgow Coma Scale; AST, aspertate aminotransferase.

**Fig 3:**
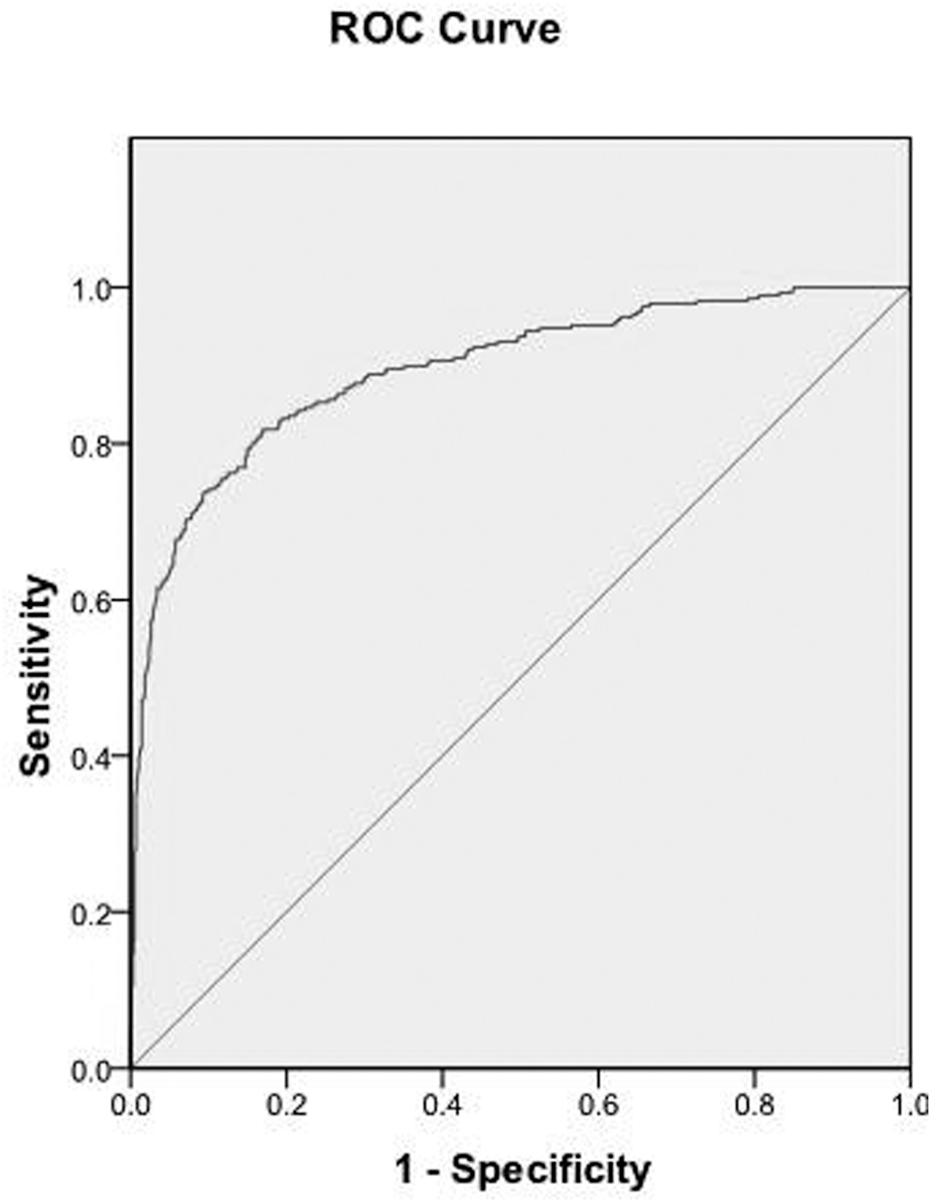
ROC curve to assess the discriminatory ability of the model predicting aspiration-related ARDS.

The ROC curve is shown in Fig. 3. The area under the curve (AUC) was 0.895 (95%CI 0.871–0.918, P<0.001). The *c* statistic value, which represents by the AUC, is considered acceptable. The model predicting aspiration-related ARDS had a better discrimination.

## Discussion

Aspiration is frequently reported in acute stroke patients with altered level of consciousness. ARDS causes severe acute respiratory failure with dynamic impairment in oxygen and carbon dioxide transfer, with the need of mechanical ventilation for high levels of supplementary oxygen [10,12,13]. Due to impaired consciousness and swallowing difficulty, acute stroke patients are at great risk for aspiration-related ARDS with high mortality. As a cause of ARDS, nonetheless, no study has comprehensively described the clinical characteristics of the disease. After the first few aspiration-related ARDS victims were encountered, we became alert to patients with similar clinical courses because of its bad prognosis and high mortality rate.

Most patients develop tachypnea and hypoxia within seven days in the course of the disease and seek for medical attention with signs of pulmonary infiltration and consolidation, then progress rapidly to respiratory failure, which requires mechanical ventilation with oxygen supplementation. The onset of tachypnea is primarily observed and most common during the prognosis of the disease. However, some patients present with fever prior to the other symptoms and subsequently develop ARDS within one week. Physicians must pay careful attention to the respiratory symptoms of fever patients, even those without signs of infectious disease, so as not to overlook the signs of ARDS. The physical findings of aspiration-related ARDS are nonspecific. Physical examination may reveal wet or dry rales on respiratory examination.

Chest radiography often shows bilateral opacities with infiltration involving in lower lobe. The information provided by plain radiograph is often limited. Compared with chest X-ray, CT scan can more accurately reflect the pulmonary lesion. On CT images, aspiration-related ARDS is characterized by diffuse ground-glass attenuation areas and inhomogenous patchy consolidations along lung marking distribution in lower areas of the lung. In the early phase, ground-glass opacity is predominantly in the lower lung lobes, which usually evidence the presence of potentially reversible. Pleural effusion and air bronchogram could also be observed. The lesion in the right lung is more severe than the left as the right principal bronchus travels straighter towards the trachea than does the left principal bronchus [2,14]. In all cases, the radiological differential diagnoses are diffuse pulmonary infiltrates, especially pulmonary edema and widespread infection. CT is not only useful for diagnosis, but also proposed to monitor disease activity and/or severity. It is advisable that performing chest CT timely is helpful for identifying disease early and prompting proper treatment to rescue our patients [15,16,17].

Earlier recognition of patients with or at high risk for developing aspiration-related ARDS is integral to any strategy targeting early intervention. Following a witnessed aspiration, turning the patient’s head laterally, raising up the bed and oral and pharyngeal suction may reduce further aspiration [7]. Lung protective strategies of mechanical ventilation remain the only therapies with an established survival advantage [10,18,19]. Though current treatment strategies for aspiration-related ARDS are unsatisfactory, the importance of early diagnosis and treatment should be emphasized.

Aspiration is relatively common in the older patients who have diminished cough reflex, which increases the incidence of dysphagia and gastroesophageal reflux [20,21]. The NIHSS score is increasingly being used as the measured of stroke severity. Risk for aspiration is increased in these patients due to the decreased bulbar reflexes and the absence of protective reflexes, which is related to the severity of the stroke [22–24]. The GCS score is generally being used as the measure of the consciousness level. The disease has a propensity to develop in patients with lower level of consciousness and shows rapid progression in its course with high mortality [25,26]. Most stroke related aspiration are believed to result from dysphagia due to neurological injury and subsequent aspiration of oropharyngeal material or gastric content [27,28]. Prior studies shown that dysarthria could cause mental stress and might increase the secretion of gastric acid [4,20]. Stress-related mucosal disease and ulcers could be the cause of aspiration of acid [29,30]. Hyperglycemia after stroke is a manifestation of the stress response [31]. Although diabetes is not susceptible to aspiration-related ARDS in analysis, stress-hyperglycemia is a part of risk assessment for the disease. Acute stroke is regularly accompanied by dysphagia, impaired consciousness and other factors caused insufficient nutritional intake, which may lead to low hemoglobin, hypoalbuminemia, and hyponatremia. Due to many reasons causing elevated AST, the association needs further research.

Some potential limitations should be noted. Firstly, the analysis was retrospective. The clinical practice could be changed substantially or that the associations between the demographic and clinical factors predictive of aspiration-related ARDS would differ currently. However, we consecutively enrolled every participant admitted with acute stroke and collected the hospitalisation medical records as completely as possible. Secondly, the potential weakness was lack of data specific to each aspiration event. The volume or pH of aspirated material and the frequency of aspiration were rarely quantified. These factors are commonly used to predict outcomes of pulmonary aspiration [7,8]. Thirdly, the patient’s past or present medical history or concomitant medication, such as corticosteroids at admission, was not taken into account in the present analysis. It was the possible confounding parameter, which might have effect on developing the disease and influencing the progress.

## Conclusions

Aspiration-related ARDS in acute stroke patients is a problem that particularly leads to long hospitalization, poor quality of life, and it is often misdiagnosed resulting in high mortality. The retrospective study describes the clinical manifestations, imaging features, and risk factors for aspiration-related ARDS in acute stroke patients, all of which are readily available in clinical settings. Patients with marked disturbance of consciousness are prone to occur to aspiration-related ARDS. It is conceivable that performing chest radiological image timely may early detect the disease and prompt strategy to improve the outcome. Early detection of these patients may improve the probability of continuing to live.
